# An Incidental Diagnosis of Rheumatic Mitral Stenosis and Secundum Atrial Septal Defect (Lutembacher's Syndrome) in a Young Woman

**DOI:** 10.1155/2019/9402987

**Published:** 2019-10-30

**Authors:** Md Mashiul Alam, Md Fakhrul Islam Khaled

**Affiliations:** ^1^Jahurul Islam Medical College Hospital, Bhagalpur, Bajitpur, Kishoregonj, Bangladesh; ^2^University Cardiac Center, Bangabandhu Sheikh Mujib Medical University, Room No-418, D Block, Dhaka, Bangladesh

## Abstract

Lutembacher's syndrome is a rare cardiovascular defect comprising of mitral stenosis and atrial septal defect. A combination of acquired mitral stenosis and congenital atrial septal defect is the most well-recognized pattern. As atrial septal defect acts as a pressure relieving gateway, signs and symptoms of mitral stenosis may be attenuated and/or delayed in such patients. We have presented a case with Lutembacher's syndrome that was incidentally diagnosed as having such defect during outpatient check-up for upper respiratory infection.

## 1. Introduction

Lutembacher's syndrome (LS) is a rare cardiovascular anomaly defined as any combination of congenital or iatrogenic atrial septal defect (ASD) with congenital or acquired mitral stenosis (MS) [1]. As mentioned, it can happen rarely, and according to the American Heart Journal, a lone person with this combination of syndrome can be found out of 1 billion people [2]. People with mitral stenosis has 0.6% to 0.7% chance of having congenital ASD while congenital MS is a rare entity accounting for only 0.6% of congenital heart disease [3]. Hence, Lutembacher's syndrome is common in the form of congenital ASD with acquired MS due to rheumatic heart disease. However, in the current era of percutaneous transluminal mitral commissurotomy (PTMC) in a patient with rheumatic MS, iatrogenic ASD due to transseptal puncture is more common than congenital ASD, where it is named as iatrogenic Lutembacher's syndrome [3]. Assuming uniform incidence of ASD, worldwide incidence of LS is more like in the region where incidence of rheumatic mitral stenosis is more such as in Southeast Asia or sub-Saharan Africa, and at the same time, this combination is rare in the western world where rheumatic mitral stenosis is uncommon. In 1916, French physician Rene Lutembacher first described the comprehensive account of these two defects in a 61-year-old woman. We have presented a case of a 25-year-old woman who was seen in the outpatient department due to simple flu-like illness and eventually diagnosed as having Lutembacher's syndrome.

## 2. Case Report

A 25-year-old young woman presented to the outpatient medicine department with the complaints of dry cough which was relentless for 1 month provisionally diagnosed as upper respiratory tract infection. On enquiry, the patient revealed that she had shortness of breath during cold seasons suggestive of bronchial asthma. She had no childhood illness indicating rheumatic heart disease. There was no significant past medical or surgical illness. She was married for two years but did not conceive yet. Her immunization history cannot be elicited as she could not remember them all. Her family members were in good health, and there was no worth mentioning illness that runs in the family. The patients' menstrual cycle was regular with an average menstrual flow. She was of a lower middle class socioeconomic status family. No family member had been suffering from any significant illness.

General examination revealed she was ill looking with mild tachypnea. Her heart rate was 90 beats per minute which was regular in rhythm; blood pressure was 80/60 mmHg; JVP was not raised. There was no anemia or edema. Her nasal mucosa was congested indicating upper airway infection.

Precordial examination showed normal apex beat in the left 5^th^ intercostal space with little bit lateral displacement. There was palpable p2 with left parasternal heave but no palpable thrill. Intensity of the 1^st^ heart sound was normal. The second heart sound was loud over the pulmonary area but not definitely splitted. There was an ejection systolic murmur over the pulmonary area with no radiation or respiratory variation.

Respiratory system examination revealed a respiratory rate of 22 breaths per minute. There was no crepitation or rhonchi.

**Figure 1:**
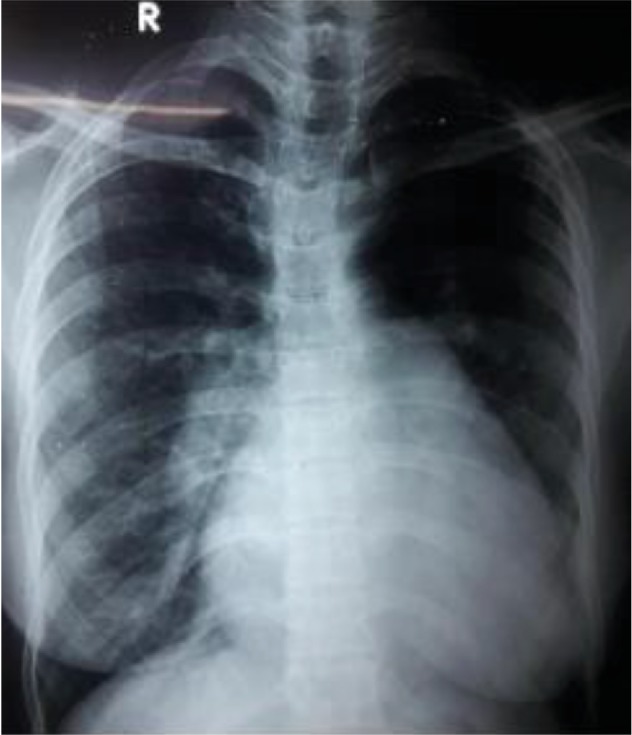
Chest X-ray P/A view showing bulged pulmonary conus, cardiomegaly, and prominent right pulmonary artery.

As our patient was being investigated for her illness, her chest X-ray revealed cardiomegaly with RV type apex, pulmonary conus was full and bulged, and right pulmonary artery was dilated (Figure 1). ECG showed right bundle branch block with right axis deviation.

**Figure 2:**
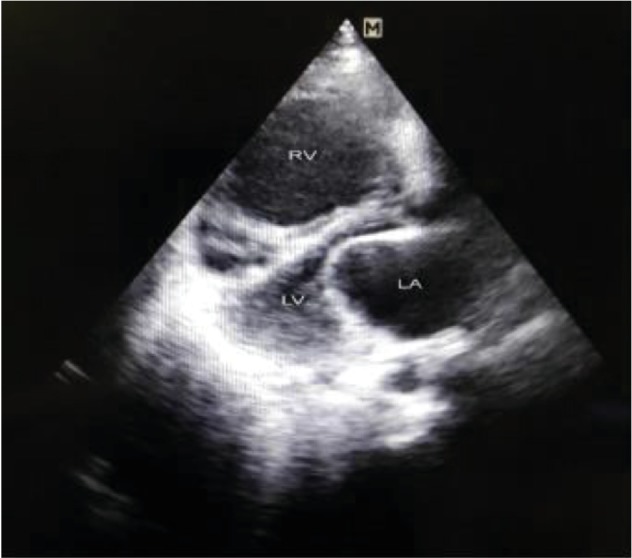
PLAX view 2D echocardiogram picture showing dilated RV and restricted MV opening.

Subsequently, her echocardiogram was done which depicted hugely dilated right atrium (RA) and right ventricle (RV) with restricted opening of the mitral valve (Figure 2).

**Figure 3:**
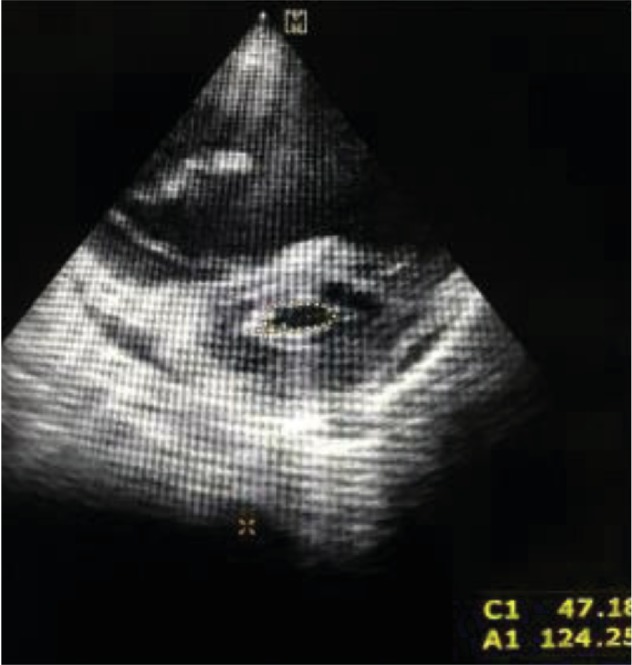
2D echocardiogram-guided planimetry showing MVA is 1.24 cm^2^.

Mitral valve planimetry showed the mitral valve area (MVA) was 1.24 cm^2^ with thickened calcified mitral valves and mild subvalvular changes (Figure 3). But the mean pressure gradient across the MV was 5 mmHg, and the MVA according to the pressure half time (PHT) method was 3.77 cm^2^. Left atrial (LA) diameter in M mode echo was 32.73 mm.

**Figure 4:**
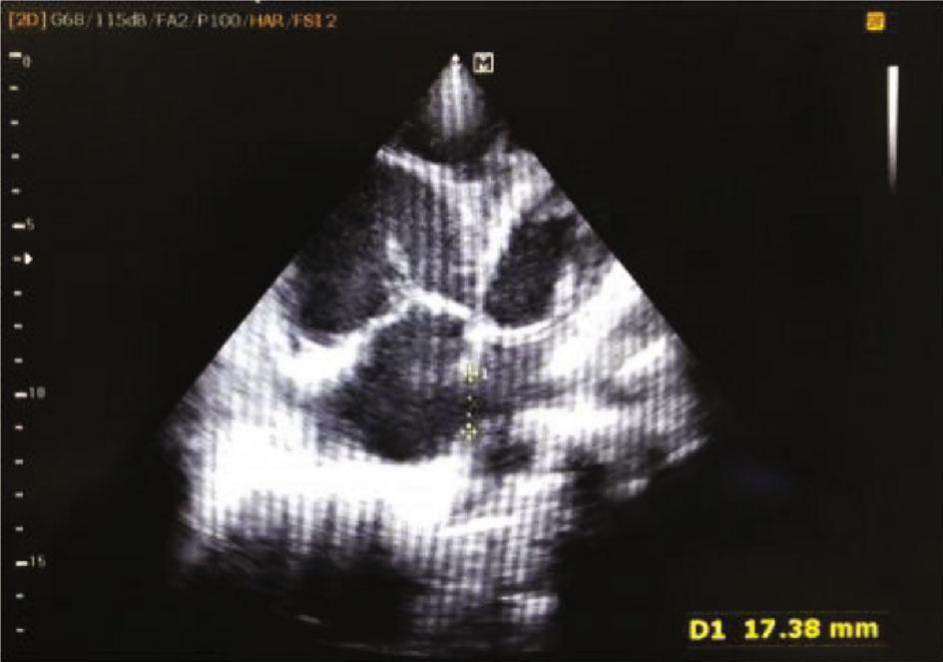
The A4CV showing RA and RV dilated compromising a left-sided heart view and ASD measuring 17.38 mm.

There was an atrial septal defect measuring 17.38 mm in the apical 4 chamber view (A4CV) (Figure 4) and 13.43 mm in the subcostal view. RV was dilated with a diameter of 55 to 60 mm at the base in the A4CV. Trabecula in RV was prominent. All rims of ASD were adequate (more than 5 mm).

The main pulmonary artery (PA) and right ventricular outflow tract (RVOT) just before the pulmonary valve (PV) were mildly dilated with 29 mm and 33 mm in diameter, respectively.

Color Doppler echocardiogram showed a mosaic flow across the mitral valve (MV) towards the LV apex during LV diastole but no regurgitation during systole. There was a butterfly-like laminar flow across the intra-atrial septum (IAS) in subcostal and A4C views.

Continuous wave Doppler across the tricuspid valve (TV) that revealed a maximum velocity through TV was 3.65 m/s. Hence, measured PASP according to the Bernoulli equation was 53.31 + 5 mm Hg = 58.31 mmHg.

A thorough study with transesophageal echo and cardiac cath could not be done due to unavailability in our center. So the patient was properly counselled about her disease condition, and she was treated with rheumatic heart disease prophylaxis, low dose of diuretics along with treatment for her presenting ailment. Furthermore, she was referred to a tertiary level hospital where all the facilities for subsequent evaluation and treatment were available. Eventually, she was undergone mitral valve replacement with metallic prosthetic valve and patch closure of ASD at a higher center. The patient had severe mitral stenosis and ASD secundum; therefore, intervention is required to prevent rising pulmonary artery pressure by increased flow through the right-sided heart, and she had occasional shortness of breath as well which she thought might be due to bronchial asthma. Though interventional treatment of LS is an option nowadays, our patient had undergone surgical repair as experience regarding percutaneous treatment is inadequate in our centers.

## 3. Discussion

Lutembacher's syndrome is one of the rare cardiovascular syndrome more commonly presents as acquired MS with congenital ASD [2]. As both ASD and MS are more frequent among female patients, this syndrome is also commonly found in female population [4]. There are several reports of familial cases of Lutembacher's syndrome, but they are isolated observations [5, 6].

Initially, it was postulated that high LA pressure due to mitral stenosis stretches open the patent foramen ovale causing left to right shunt or atrial septal defect [7]. But according to the current definition of this syndrome ASD could be acquired or congenital. High LA pressure causing ASD is further supported by some observations by Ansari and Maron [8] in 1997 when they published a case of mitral valve replacement in a 49-year-old woman. After the valve replacement, left to right shunt was no longer detectable. Similar observation was reported by Ananthasubramaniam et al. in 2001 [9].

As MS and ASD occur together, hemodynamic expression and clinical features depend on the interplay of several conditions, namely, size of ASD, severity of MS, and distensibility of RV [4]. Increased LA pressure due to mitral stenosis in Lutembacher's syndrome finds ASD as a second exit for LA blood as a result severity of MS abates by shunting of blood from LA to RA and size of ASD can regulate blood shunt consequently deleterious effect of rising LA pressure. In case there is no ASD, increased atrial pressure due to MS causes blood to find its way to pulmonary veins and hence causes pulmonary congestion more frequently and easily. In Lutembacher's syndrome, ASD prevents back pressure to pulmonary vasculature and congestion. Likewise, features related to lung congestion such as orthopnea, paroxysmal nocturnal dyspnea, hemoptysis, and pulmonary edema are delayed or attenuated. In contrast, these symptoms are substituted by features of low cardiac output, namely, fatigue, low blood pressure.

The ameliorating effect of atrial septal defect in Lutembacher's syndrome was reported by Lutembacher's original article in 1916; a 61-year-old female patient had been pregnant for seven times despite this cardiovascular anomaly [10]. Perloff revealed a case of 74-year-old woman who had gone through 11 pregnancies and survived until advance age in spite of Lutembacher's syndrome [11].

Though ASD ameliorate the severity features of MS, increased blood flow through ASD due to stenosis in mitral valve causes more blood shunted to right-sided heart predisposing atrial fibrillation and right ventricular failure [12]. In late advanced stage, such patients develop pulmonary hypertension and heart failure with poor prognosis [13]. Nevertheless, correcting this dual problem by surgical closure of ASD and mitral valve replacement bears a good prognosis and prolongs life. In recent days, an interventional technique is frequently judged to be implemented if feasible by doing percutaneous transluminal mitral commissurotomy (PTMC) and closing ASD by the Amplatzer device [14].

Hemodynamic decompression of LA pressure builds up by mitral stenosis has a considerable effect on the physical examination of a patient with Lutembacher's syndrome. The clinical examination finding of pure MS are attenuated or may be absent. Loud first heart sound, opening snap, and middiastolic murmur with presystolic accentuations are usually not heard. Generally, a continuous murmur is heard because of high LA to low RA pressure difference across ASD which persist during the entire cycle [15]. As more blood is shunted in contrast to isolated ASD, increased pulmonary blood flow across the pulmonary valve produces loud ejection systolic murmur over the pulmonary area. A holosystolic murmur over the left lower sternal edge may accompany as a finding, while pulmonary hypertension may cause a regurgitant tricuspid lesion.
